# Doped Carbon Dots for Sensing and Bioimaging Applications: A Minireview

**DOI:** 10.3390/nano8050342

**Published:** 2018-05-18

**Authors:** Timur Sh. Atabaev

**Affiliations:** Department of Chemistry, School of Science and Technology, Nazarbayev University, Astana 010000, Kazakhstan; timuratabaev@yahoo.com or timur.atabaev@nu.edu.kz

**Keywords:** carbon dots, doping, fluorescent propertum dots with pH- and thies, sensing, bioimaging

## Abstract

In the last decade, carbon dots (C-dots, CDs) or carbon quantum dots (CQDs) have attracted a considerable amount of attention from the scientific community as a low cost and biocompatible alternative to semiconductor quantum dots. In particular, doped C-dots have excellent fluorescent properties that have been successfully utilized for numerous applications. In this minireview, we overview the recent advances on the synthesis of doped C-dots derived from carbon-rich sources and their potential applications for biomedical and sensing applications. In addition, we will also discuss some challenges and outline some future perspectives of this exciting material.

## 1. Introduction

**Figure 1:**
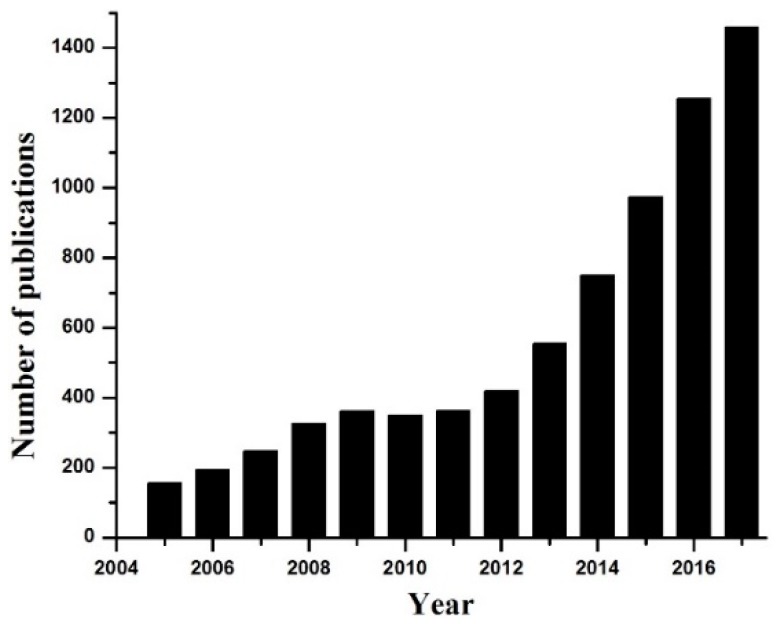
Number of published works according to the Scopus search (keyword “carbon dots”).

Carbon dots were first discovered in 2004 during single-walled carbon nanotubes purification [1]. These C-dots can be described as quasi-spherical particles with sizes below 10 nm. Figure 1 shows that the number of scientific publications related to C-dots rapidly raised starting from 2005. The increased interest in these materials is related to several advantages of C-dots, such as carbon source abundance, simple and low-cost synthesis process, biocompatibility, and excellent fluorescent properties. In addition, C-dots are chemically stable, inert, form a stable colloidal solution, and are highly resistant to photobleaching compared to traditional fluorescent organic dyes and semiconductor quantum dots. Therefore, the fluorescent properties of C-dots are widely utilized for sensing [2,3,4], biomedical imaging [5,6,7], catalysis [8,9], energy research [10,11], etc. A number of review articles related to the synthesis and various applications of C-dots were published recently [1,4,11]. On the other hand, a focused overview of doped C-dots for potential biomedical and sensing applications is still not available in the literature. Therefore, in this minireview, we briefly discuss the synthesis and fluorescent properties of doped C-dots obtained from carbon-rich sources. Next, we review recent representative works related to the sensing and biomedical applications of doped C-dots. Finally, we will provide some future perspectives and highlight the importance of doping strategy for the development of advanced multifunctional nanoprobes.

## 2. Synthesis and Optical Properties of Doped C-Dots

Various synthetic methods such as chemical etching [12], electrochemical carbonization [13], laser ablation [14], microwave irradiation [15], and hydrothermal/solvothermal [16,17] methods have been widely employed for the preparation of fluorescent C-dots. Considering the enormous potential of C-dots for numerous applications, it is extremely necessary to develop their large-scale synthesis. From this point of view, the hydrothermal synthesis has become the most popular method due to its simplicity, low-cost, and high efficiency. In this method, one should simply heat the water and carbon-rich compounds (sugars, organic molecules) in a tightly closed vessel to initiate the carbonization process.

**Figure 2:**
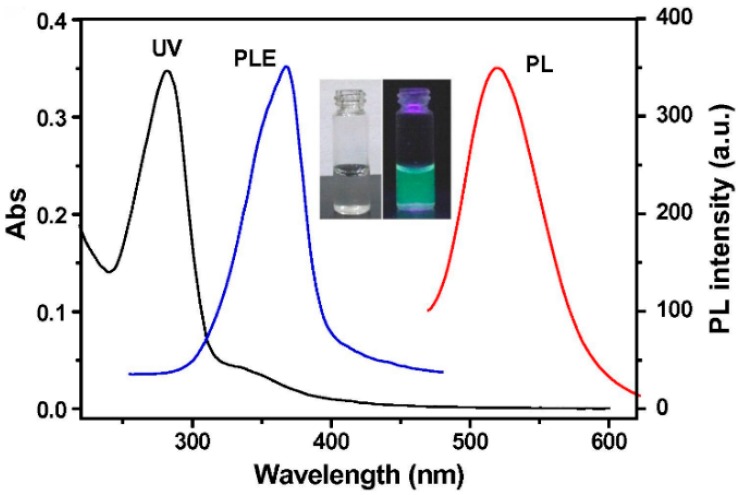
UV–vis absorption (UV), PL excitation (PLE), and PL emission (PL) spectra of doped C-dots. Reprinted with permission from reference [15].

The size of C-dots is usually controlled by the concentration of reactants, temperature of reaction, time, surfactants, additives, etc. For example, the hydrothermal method was used to prepare highly fluorescent C-dots from orange juice [18], glucose [19], banana juice [20], citric acid [21], etc. To date, the majority of the works report the fabrication of blue emitting C-dots with excitation-dependent emission spectra. However, the emission spectra of C-dots can be tuned by doping with some elements such as nitrogen (N), sulfur (S), phosphorus (P), boron (B), or their combinations [22,23,24,25,26]. It has been shown that doping of C-dots leads to the fluorescence enhancement and shift of emission spectra. For example, fluorescent C-dots with green emission spectra (Figure 2) were reported and employed for sensing and cell imaging [15,27].

C-dots typically show high absorption in the UV range (~287 nm) which can be attributed to n-π* transition of the C=O and π-π* of the C=C bond. The origin of fluorescence in C-dots was not fully clarified yet, thus, various interpretations can be found in the literature. For example, the origin of fluorescence was attributed to the electron-hole pairs recombination in small sp^2^ carbon clusters that are localized within a sp^3^ matrix [28], various surface states and defects [29], surface groups [30], quantum effects (size dependent) and surface passivation [31]. Recently, Wang and coworkers used ultrafast spectroscopy to investigate the origin of green fluorescence in C-dots and graphene quantum dots [32]. According to their results, the green fluorescence originated from several carbon atoms located on the edge of carbon backbone and some functional groups (carbonyl, carboxyl). Different emission centers and traps can be created with the introduction of dopant atoms into C-dots. As a result, the emission spectra can be tuned to green and even red spectra [33]. However, in some cases dopant elements are located on the surface of C-dots only [34]. Thus, the competition between the optical centers, surface states, and traps is mainly responsible for the emission spectra of doped C-dots. The exact mechanism of fluorescence shifting in doped C-dots still under investigation, thus, further discoveries are expected in near future. Nevertheless, the fluorescent properties of doped C-dots were already utilized for numerous technological applications [2,10,35]. Therefore, the practical usage of doped C-dots for biomedical and sensing applications will be discussed in the following sections.

## 3. Doped C-Dots for Sensing Applications 

**Figure 3:**
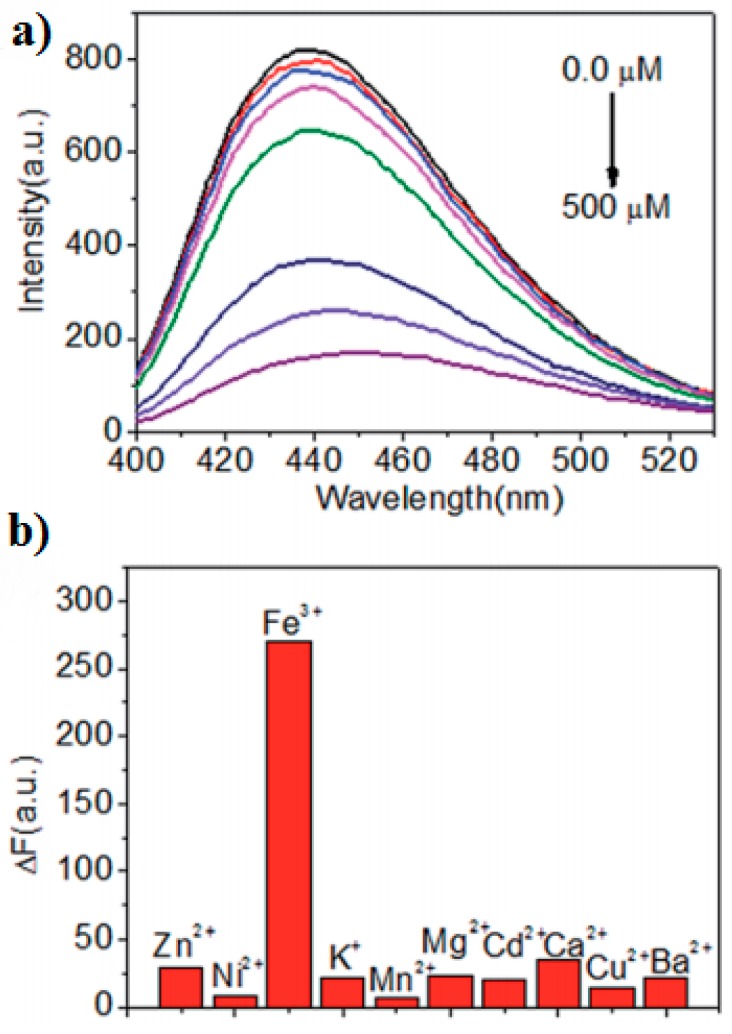
(**a**) The emission spectra of sulfur-doped C-dots in the presence of Fe(III) at different concentrations; (**b**) the fluorescence quenching (selectivity) of sulfur-doped C-dots in the presence of different metal ions. Reprinted with permission from reference [26].

Analysis of the fluorescent properties of doped C-dots were widely employed for the detection of metal ions, molecules, temperature, pH range, etc. In particular, several research groups utilized doped C-dots for the quantitative detection of the Fe(III) ions [26,36,37]. The fluorescence intensity of doped C-dots decreased monotonically (Figure 3a) as the Fe(III) concentration increased. It was suggested that fluorescence quenching (Figure 3b) occurs due to strong coordination of Fe(III) to the oxygen-rich or amine groups on the surface of doped C-dots. 

It is well-known that the environmental pollution with mercury (Hg^2+^) and lead (Pb^2+^) can raise serious health issues. Recently, doped C-dots were also used as sensors to detect these ions in water. For example, Liu and coworkers reported the preparation of ultrasensitive C-dots for selective detection of Hg^2+^ [38]. Prepared C-dots have been applied for Hg^2+^ detection in tap, lake, and river water with an excellent detection limit of 1 fM. In another study, authors prepared sensitive Hg^2+^ nanosensor based on nitrogen-doped fluorescent C-dots with a detection limit of 0.23 µM [39]. Pb^2+^ ions were also fluorescently detected by C-dots. For example, C-dots prepared from chocolate have a detection limit of 12.7 nM [40]. Jiang et al. used a microwave method to prepare nitrogen-doped C-dots with a similar detection limit of 15 nM [41]. It was suggested that the quenching mechanism may occur due to the nonradiative electron transfer from the excited states to the electronic orbitals of Hg^2+^ and Pb^2+^. On the other hand, chelation between Pb^2+^ and hydroxyl groups on the surface of C-dots can be another reason for fluorescence quenching [40]. In addition, fluorescent properties of C-dots were also utilized for detection of other metal ions such as Ag^+^ [42], Cu^2+^ [43], Zn^2+^ [44], and Cr^6+^ [45]. Thus, the selectivity of C-dots to some toxic metal ions can be compromised by the presence of other metal ions. To resolve this issue, one can perform the surface modification of C-dots with some receptors for selective detection of metal ions. 

**Figure 4:**
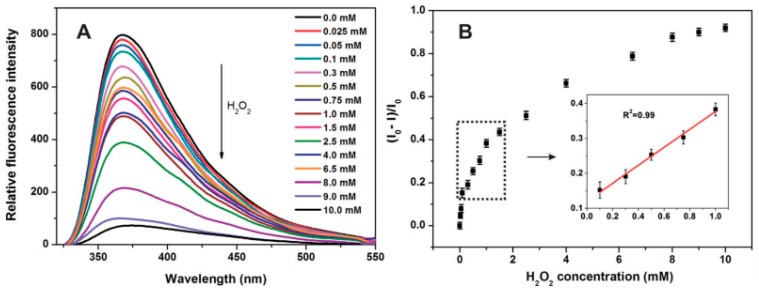
(**A**) The emission spectra of boron-doped C-dots in the presence of H_2_O_2_ at different concentrations; (**B**) the fluorescence response of boron-doped C-dots to the H_2_O_2_ at different concentrations. Reprinted with permission from reference [47].

The fluorescent properties of C-dots were also widely utilized for the detection of various molecules. For example, fluorescence quenching of C-dots dispersed in dimethyl sulfoxide (DMSO) was reported with acetone addition with detection limit up to 1:10^–7^ M ratio (DMSO:acetone) [46]. Shan and coworkers showed that boron-doped C-dots demonstrated an excellent ability to detect the hydrogen peroxide H_2_O_2_ and glucose molecules [47]. In this work, the charge transfer between boron and H_2_O_2_ trigger the fluorescence quenching of C-dots, which allow the quantitative detection of hydrogen peroxide in the range of 0.1 to 1.0 mM (Figure 4). In another study, cyclic voltammetry was employed for sensitive detection of glucose with the help of nitrogen-doped C-dots with a detection range of 1–12 mM [48]. Furthermore, nitrogen-doped C-dots were commonly utilized for fluorescent detection of pyridine [49], dopamine [50], amoxicillin [51], microRNA [52], catechol [53], pH and temperature [54], etc. Taking into account such a diverse range of doped C-dots applications, future studies should concentrate on the following open issues: (a) the origin of fluorescence shifting in doped C-dots; (b) the precise localization of doped elements in C-dots; and (c) the role of doped elements in fluorescence sensing.

## 4. Doped C-Dots for Biomedical Applications

**Figure 5:**
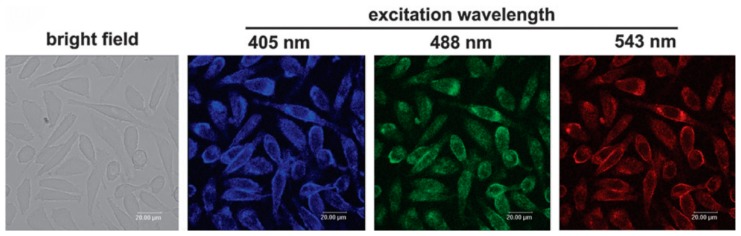
Confocal microscopy images of L929 cells incubated with C-dots under different excitation wavelengths. Reprinted with permission from reference [58].

Compared to conventional organic dyes, C-dots are not suffering from photobleaching and photodegradation. Therefore, C-dots demonstrated a great potential for cell imaging and labeling as a biocompatible fluorescent nanomaterial. For example, numerous studies reported the cell imaging potentials of doped C-dots [55,56,57]. Due to excitation-dependent fluorescence emission, C-dots can be used as a multicolor nanoprobe that can be excited with different excitation wavelengths. Zhai and coworkers showed that C-dots incubated with L929 cells (Figure 5) could emit blue, green, and red fluorescence upon 405, 488, and 543 nm excitation, respectively [58].

**Figure 6:**
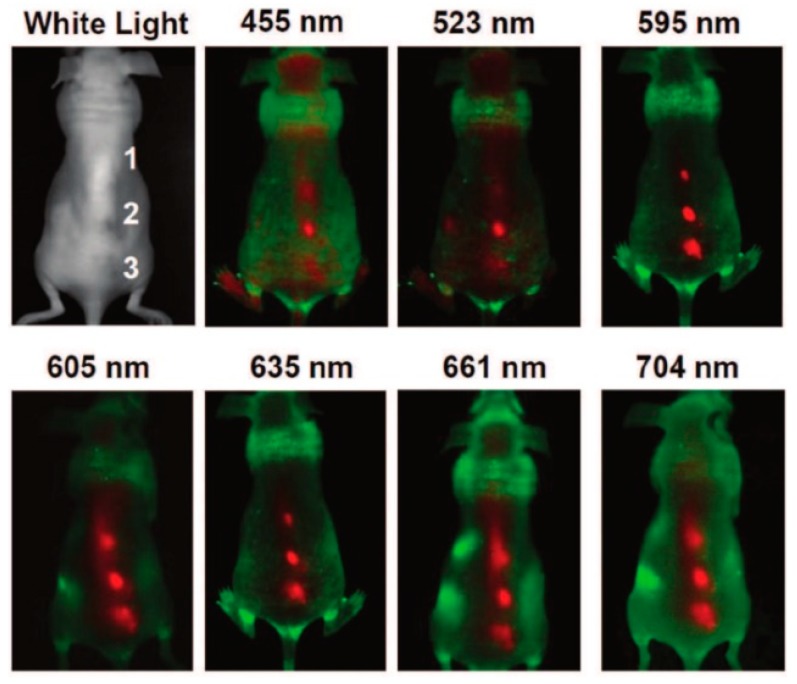
In vivo fluorescence images of C-dots injected into nude mouse. Reprinted with permission from reference [60].

As it was demonstrated by several studies, the fluorescent properties of C-dots can be also efficiently applied for in vivo imaging [59,60]. Excitation-dependent C-dots were injected into a mouse and fluorescent images were collected at different excitation wavelengths (Figure 6). It was found that longer wavelengths (595 nm and beyond) demonstrated better signal-to-background contrast.

Recently, enormous attention has been paid to multifunctional nanoprobes that combine several imaging modalities. In this regard, one can create thin carbon layer coating on the surface of paramagnetic nanoparticles such as Gd_2_O_3_ [61]. Obtained core-shell nanostructure has a longitudinal relaxivity of r_1_ = 4.23 ± 0.13 mM^–1^ s^–1^ which is suitable for T_1_-weighted MRI. At the same, C@Gd_2_O_3_ core–shell nanostructure was also useful for optical imaging of L929 cells thanks to the fluorescent properties of the thin carbon layer. In addition, carbon thin coating can be useful as a protective layer to prevent the leaching of Gd ions into a surrounding environment. On the other hand, paramagnetic metals can be also directly doped into C-dots during the synthesis process. Paramagnetic gadolinium (Gd)-based complexes are widely used as ‘positive’ T_1_-weighted contrast agents in radiology [62,63]. Therefore, Gd-doped C-dots were studied extensively for both magnetic resonance imaging (MRI) and cellular imaging.

**Figure 7:**
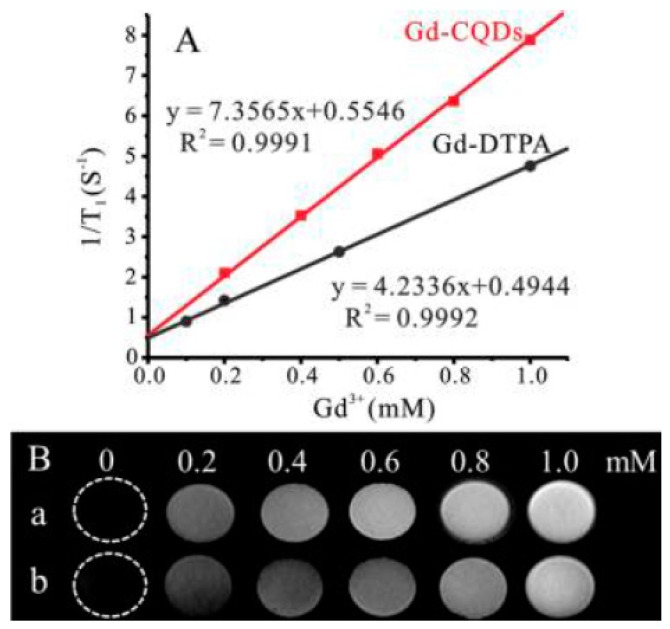
(**A**) T_1_-weighted relaxivity rates of Gd-doped C-dots and Gd-DTPA as a function of Gd^3+^ concentration; (**B**) MRI images of (a) Gd-doped C-dots; and (b) C-Gd-DTPA taken at different concentrations. Reprinted with permission from reference [65].

Chen and coworkers prepared Gd-doped C-dots by the direct calcination of gadopentetic acid (Gd-DTPA) in the air [64]. It was found that prepared Gd-doped C-dots exhibited excellent fluorescence quantum yield ~19.7%. Furthermore, these Gd-doped C-dots have high longitudinal relaxivity r_1_ = 5.88 mM^–1^ s^–1^ at 7 T, much higher than commercially available Gd-DTPA (r_1_ = 3.10 mM^–1^s^–1^) under the same conditions. Xu and coworkers used a facile hydrothermal method to prepare Gd-doped C-dots with longitudinal relaxivity r_1_ = 7.36 mM^–1^ s^–1^ at 1.2 T [65]. The imaging site becomes brighter with increasing of Gd concentration (Figure 7), and it obviously highlights that Gd-doped C-dots can be utilized as ‘positive’ MRI contrast agents. Wang and coworkers used paramagnetic manganese Mn ions as a cheaper and less toxic alternative to Gd(III) [66]. According to results, Mn-doped C-dots have the longitudinal relaxivity of r_1_ = 7.28 mM^–1^ s^–1^ at 1.2 T, which is comparable to Gd-doped C-dots.

## 5. Summary and Future Outlook

Doped C-dots can be a versatile material for future biomedical and sensing applications thanks to the unique fluorescent properties, excellent biocompatibility, and high aqueous stability. The fabrication procedure of doped C-dots is quite simple and low-cost due to the wide selection of cheap carbon-rich sources. Furthermore, some fabrication procedures allow the preparation of doped C-dots at relatively large amounts, typically at gram-scale. On the other hand, some issues such as the exact origin of fluorescence, the role of dopant ions, and their location in C-dots were not resolved yet. Thus, these issues should be addressed for fundamental understanding and control of the fluorescence in doped C-dots. One can consider the surface-functionalization of doped C-dots for specificity and selectivity for sensing and biomedical research. For example, C-dots conjugated with folic acid (FA) were able to distinguish the cancer cells from normal cells in a model mixture of cells thanks to a specific interaction between the FA and folate receptor molecule [67]. In a similar manner, doped C-dots can be surface-functionalized for selective recognition of specific ions, molecules, or even cells. The formation of mesoporous surface-functionalized C-dots that can be potentially used in controlled drug delivery can be also another breakthrough in this research area. The existence of limited studies on the development of doped C-dots for T_2_-weighted MRI (‘negative’ contrast agents) can be also considered as another unresolved problem. In particular, Dy and Ho ions with high magnetic moments can be introduced into C-dots to prepare ‘negative’ contrast agents for MRI and optical imaging [68,69]. As one can see, doped C-dots can be a cheap and promising material for various applications such as optics, sensors, biomedicine, energy, etc. Therefore, we strongly believe that the number of studies on doped C-dots will continue to grow in future.
